# Performance-normalized life cycle assessment of calcined and mechanochemically activated clays in Australia as cement substitutes

**DOI:** 10.1007/s11356-026-38183-z

**Published:** 2026-09-04

**Authors:** Ishan Basnayaka, Chamila Gunasekara, David Law, Roshan Jayathilakage, Sujeeva Setunge

**Affiliations:** Civil & Infrastructure Engineering, RMIT University, GPO Box 2476, Melbourne, Vic 3001 Australia

**Keywords:** Performance-based LCA, Calcination, Mechanochemical activation, Low-carbon binder, Reclaimed waste clay, Sustainable construction materials

## Abstract

In response to the decreasing availability of high-grade kaolinitic clays, there is a growing shift toward using natural waste clays as supplementary cementitious materials. The performance of these waste clays is generally evaluated based on either strength or environmental impact, but the combination of both is rarely considered. This study undertakes a strength-normalized life cycle assessment to understand the interaction between these two parameters and utilizes the output to identify the most suitable activation process. The study used five Australian natural waste clays activated by calcination (600–900 °C) and high-shear mechanical grinding, replacing 30% of the general-purpose cement by mass with the activated clay. The environmental impacts were normalized to MPa using the Australian life cycle impact assessment framework. Results revealed that calcined binders achieved up to 18.6% lower global warming potential than general-purpose cement. However, burden shifting can be observed, with mechanochemically activated clays exhibiting up to 255% higher abiotic depletion potential, primarily due to increased electricity consumption and the use of steel grinding media. Sensitivity analysis showed that reducing transport distance by 100 km decreased resource depletion by 3.0%, human health impacts by 0.7%, and ecosystem damage by 0.5%. Using projected renewable energy sources and alternative fuel sources for activation reduced environmental impacts by up to 18%. These results underscore that the suitability of activated natural waste clays depends not only on the percentage of cement replacement but also on clay type, activation method, energy source, and transport logistics.

## Introduction

Concrete is the most widely utilized construction material globally, while cement remains the highest-consumed industrial product by mass (Scrivener et al. 2018). The environmental footprint of concrete is substantial, primarily due to the production of Portland cement, which accounts for 5–7% of global CO₂ emissions (Bianco et al. 2021). The calcination of limestone and subsequent clinker production are highly energy-intensive processes that release significant quantities of greenhouse gases (Antunes et al. 2021). Furthermore, cement manufacturing necessitates extensive raw material extraction, particularly of limestone and clay, contributing to land degradation and biodiversity loss (Habert et al. 2020).

In Australia, 97% of clinker production is powered by coal and gas, producing approximately 975 kg of CO₂ emissions per ton of clinker produced (Rouch 2023). Of these emissions, 55% originate from the calcination of limestone, while 26% are attributed to the energy required for the provision of heat (Rouch 2023). New supplementary cementitious materials (SCMs) partially replacing cement are being explored to decarbonize the construction sector, while SCMs, including fly ash, slag, and activated clays, not only reduce carbon emissions but can also enhance or maintain the mechanical properties of concrete (Lothenbach et al. 2011; Provis and Bernal 2014; Vargas and Halog 2015). Due to the declining availability and regional limitations of industrial by-products such as fly ash, slag, and other conventional SCMs, activated clays have emerged as a promising alternative. This is due to their widespread and abundant availability across various geographies. For example, the global reserves of kaolin alone were estimated at 32 billion tons in 2022 (Cai et al. 2023; Fitos et al. 2015; Souri et al. 2015; Viscarra Rossel 2011).

There are two primary methods for activating clays: calcination and mechanochemical activation (Jayathilaka et al. 2024; Jayathilakage et al. 2025a, b). Schieltz and Soliman (1964) state that the theoretical heat required to produce metakaolin from pure kaolinite in calcination is 1145 kJ/kg, corresponding to only 64% of the thermal energy needed to produce clinker. While this value does not reflect the full complexity of actual production for either process, it clearly illustrates a difference in energy demand. The temperature required for the dehydroxylation of clay minerals is significantly lower than that for the decomposition of limestone. As a result, this lower temperature requirement helps reduce emissions associated with heat provision. Dumani and Mapiravana (2024) demonstrated that replacing 30% of ordinary Portland cement (OPC) with calcined clay resulted in a 17.3% reduction in CO₂ emissions. Similarly, Bumanis et al. (2022) state a 15% reduction in GWP with a 25% cement replacement using waste kaolin. A cement binder studied by Sadok et al (2022) using 25% calcined sediments and 75% cement resulted in a 17.8% reduction in CO_2_ production and a 17.22% reduction in energy demand. These reductions contributed to cuts of 10% in human health-related, 7% in ecosystem quality-related, 18% in climate change-related, and 19% in resource-related impact categories. Similarly, several studies have demonstrated that calcined clay-cement binders can provide environmental benefits at replacement levels of approximately 25–30%. However, their findings are mainly based on high-grade kaolin, waste kaolin, or sediment-based systems rather than naturally occurring mixed-clay sources, where a mix of different clay types is present.

Mechanochemical activation involves high-shear grinding of the material to increase fineness and specific surface area, thereby enhancing reactivity (Tole et al. 2022). This method bypasses direct fossil fuel consumption associated with calcination, relying instead on electrical energy for milling. Therefore, the GWP of mechanochemically activated clay is highly sensitive to the carbon intensity of the electricity used. Tole (2019) suggested that mechanochemical activation may present an energy-efficient alternative. However, their assertion lacks comprehensive experimental validation or quantitative evidence to substantiate the proposed energy benefits. Moreover, mechanochemical activation does not meet the energy requirement to decompose the carbonates present in the clay, further reducing CO₂ emissions (Mandrino et al. 2018). On the other hand, the manufacturing and use of grinding media such as steel balls may lead to slightly higher impacts in categories like resource depletion and human toxicity (Liu et al. 2020; Shi et al. 2022). The magnitude of these impacts depends on factors like wear rate and recycling efficiency.

In addition to the activation method, the cement replacement level also has a major influence on both environmental impacts and the strength of the binder. Previous studies have shown that activated clays help reduce CO₂ emissions by partially replacing clinker while still producing a strength activity index (SAI) greater than 1 for up to 20% replacement of cement (Al-Hashem et al. 2022; Souri et al. 2015; Tole et al. 2022). By replacing cement, SCMs directly avoid the emissions associated with limestone decomposition, which accounts for approximately 55% of total CO₂ emissions during clinker production (Rouch 2023). Therefore, an increase in replacement level leads to further reductions in emissions associated with cement binder production. However, the suitable replacement level must be selected by considering the compressive strength performance achieved at each replacement level, as this depends on the clay mineralogy and activation method.

Pillai et al. (2019) combined service-life estimation with life cycle assessment for LC^3^ concrete and reported that LC^3^ could achieve a 16–30% lower CO₂ footprint than OPC concrete of similar strength. Similarly, Jin et al. (2024) assessed LC^3^ concrete at a comparable 28-day compressive strength of 30 MPa and reported GWP reductions of 36% in the Quebec context and 46% in the French context. Furthermore, Bediako and Valentini (2022) evaluated low-grade calcined kaolin pozzolan concrete with 10–50% cement replacement, considering both compressive strength and carbon emissions using a carbon index. Their results showed that carbon emissions decreased progressively with increasing pozzolan content. However, the strength response varied with replacement level, highlighting the need to assess cement replacement using both environmental and mechanical performance criteria. However, these studies have mainly focused on concrete systems or LC^3^ formulations, in which aggregate content, limestone addition, and mix design influence the environmental assessment.

Although research is increasingly moving toward the use of natural mixed clays as supplementary cementitious materials and assessing their performance in binders, their environmental impacts remain less studied. This limits a complete evaluation of their overall performance and makes it difficult to compare their environmental benefits with those of conventional cement binders and other established supplementary cementitious materials. While the life cycle assessment (LCA) of the calcination process has been widely studied, including in LC^3^ binders, mechanochemical activation has received comparatively less attention. As a result, limited quantitative data have compared the two approaches, calcination and mechanochemical activation, under identical boundary conditions. Key questions remain around each process’s energy and carbon trade-offs, particularly when considering different power sources and their associated impacts.

Existing studies on calcined clays have primarily focused on high-purity kaolinite sources (Dumani and Mapiravana 2024). However, supply constraints and competition from industries such as ceramics and paper increasingly limit the availability of high-grade kaolinite-rich clays for use as SCMs. In response, this study uses Australian natural mixed-layer clays, a blend of clay and non-clay minerals. Their varying pozzolanic reactivity, chemical composition, and physical structure influence not only the mechanical performance of the final cementitious product but also the environmental profile of the resulting binder. Furthermore, LCA studies on activated clays remain limited, particularly within regional contexts. This highlights the need for region-specific evaluations that consider how local material characteristics and activation methods influence both mechanical performance and environmental impact under local energy conditions.

### Research significance

Supply constraints and competition from industries such as ceramics and paper increasingly limit the use of high-grade kaolinite-rich clays as SCMs. This study provides crucial insights into the environmental viability of utilizing more readily available low-grade natural or reclaimed clays. Moreover, no studies to date have compared the LCA of two activation methods (calcination and mechanochemical activation). Additionally, assessments of mechanochemical activation remain limited. This highlights an essential gap in the existing knowledge. More importantly, this study evaluates these binders using a performance-normalized LCA approach, where environmental impacts are assessed relative to the measured compressive strength of each binder. A strength-based normalization metric (per MPa) advances conventional LCA methodology beyond mass or volume-based assessments, and the results are evaluated relative to a general-purpose cement binder. This enables functionally equivalent comparisons across binder systems with varying mechanical performance. To establish the performance-based comparison, compressive strengths of each binder system are experimentally determined and used as the basis for environmental impact allocation. The analysis incorporates region-specific energy mixes, material sourcing routes, and activation pathways, addressing a critical research gap in SCM evaluations where globally generalized data are often misaligned with local conditions.

## Materials and methods

This study was conducted in two stages. First, a series of preliminary experiments was carried out to characterize the five clays. These initial studies were to determine the optimal compressive strength of each clay when used as a supplementary cementitious material after calcination and mechanochemical activation. Based on the outcomes of these experimental trials, an LCA was conducted to compare the environmental performance of each activated clay system under both calcination and mechanochemical activation pathways.

### Materials and preliminary studies

The study evaluates a binary binder composed of activated clay and cement. Five low-grade clay samples with varying mineralogical compositions were sourced from different regions across Australia. This includes two kaolinite-dominant natural mixed clay materials (Red Sealing Clay (RSC) and Blended Sealing Clay (BS)), illite-dominant Clay B (CB), kaolinite and illite mixed Hanson White Clay (HW), and montmorillonite and carbonate mixed bentonite clay (BN). The clays were oven-dried at 110 °C for 24 h to remove moisture. The dried materials were then crushed and ground through a 75-µm sieve.

Activation conditions for each sample were determined based on findings from preliminary studies aimed at optimizing the binder strength after 28 days. In the preliminary calcination study, each clay was thermally activated at temperatures between 600 and 900 °C, and the resulting binders were assessed for compressive strength after 28 days. The optimum calcination temperature was defined as the temperature that produced the highest 28-day compressive strength for each clay. This corresponded to 900 °C in calcination for BN clay, while the other clays were calcined at 600 °C. In contrast, mechanochemical activation was performed using the same grinding parameters for all five clay types, based on preliminary studies conducted on the same clay types (Table 1) (Jayathilaka et al. 2024; Jayathilakage et al. 2026a, b). Table 1 summarizes the parameters used for activation.

**Table 1:** Parameters used in activation

Calcination		Mechanochemical activation	
Ramp rate	10 °C/min	Speed	500 rpm
Final temperature	600 °C/900 °C	Duration	20 min
Dwell time	1 h	Grinding media	Steel balls
		Ball diameter	10 mm
		Ball/powder	10

Following the activation process, binder specimens were produced by substituting 30% of general-purpose cement (GPC) (compliant with AS 3972 Type GP standards) with activated clay. The 30% cement replacement level was selected based on preliminary mix design optimization conducted by the authors on the same Australian natural mixed clays. These preliminary studies evaluated different cement replacement levels and identified that a 30% replacement provided the highest 28-day compressive strength among the investigated binder compositions. Table 2 illustrates the mix design for all five clay-based binders. CC denotes the calcined clay, while MA denotes the mechanochemically activated clay. Paste mixes were prepared for each blend to compare the compressive strengths of the binders, along with a reference mix containing 100% cement. The dry blend containing activated clay and cement was mixed by hand for 3 min. After adding the water, hand mixing was continued for 2 min, and final mixing was done using a Thinky planetary vacuum mixer. A water-to-binder ratio of 0.4 (by weight) was used, and the mixes were cast into 25 mm × 25 mm × 25 mm cubic molds. After 24 h, the specimens were demolded and placed in potable water at a controlled temperature of 23 ± 2 °C for curing until 28 days.

**Table 2:** Mix design

Mix no	Mix code	Mix proportions, % by mass of binder		
		GPC	Clay	Water
1	GPC	100	0	40
2	CC_RSC/MA_RSC	70	30	40
3	CC_BS/MA_BS	70	30	40
4	CC_CB/MA_CB	70	30	40
5	CC_HW/MA_HW	70	30	40
6	CC_BN/MA_BN	70	30	40

At 28 days, the hardened specimens were subjected to compressive strength testing using a Shimadzu universal testing machine with a capacity of 50 kN. Four cubes from each mix were tested at a loading rate of 208 N/S in accordance with AS 1012.9:2014. The results are presented in Table 3.

**Table 3:** Compressive strength

Mix	Compressive strength (MPa)
GPC	57.5 (± 6.0)
CC_RSC	58.6 (± 4.6)
CC_BS	54.7 (± 3.4)
CC_CB	52 (± 2.2)
CC_HW	50.6 (± 6.6)
CC_BN	56.2 (± 3.1)
MA_RSC	53.8 (± 0.4)
MA_BS	52.4 (± 5.5)
MA_CB	41.9 (± 2.0)
MA_HW	40.0 (± 1.9)
MA_BN	36.1 (± 1.8)

### Life cycle assessment

An LCA approach was adopted to assess the environmental benefits and trade-offs of using activated clay as a cement replacement. LCA is a systematic method standardized by ISO 14040:2006 and 14044:2006 for evaluating the environmental impacts of products or processes across their life cycle. It is widely used in the construction sector to compare the environmental impacts of alternative green materials.

#### Goal and scope of the study

**Fig. 1:**
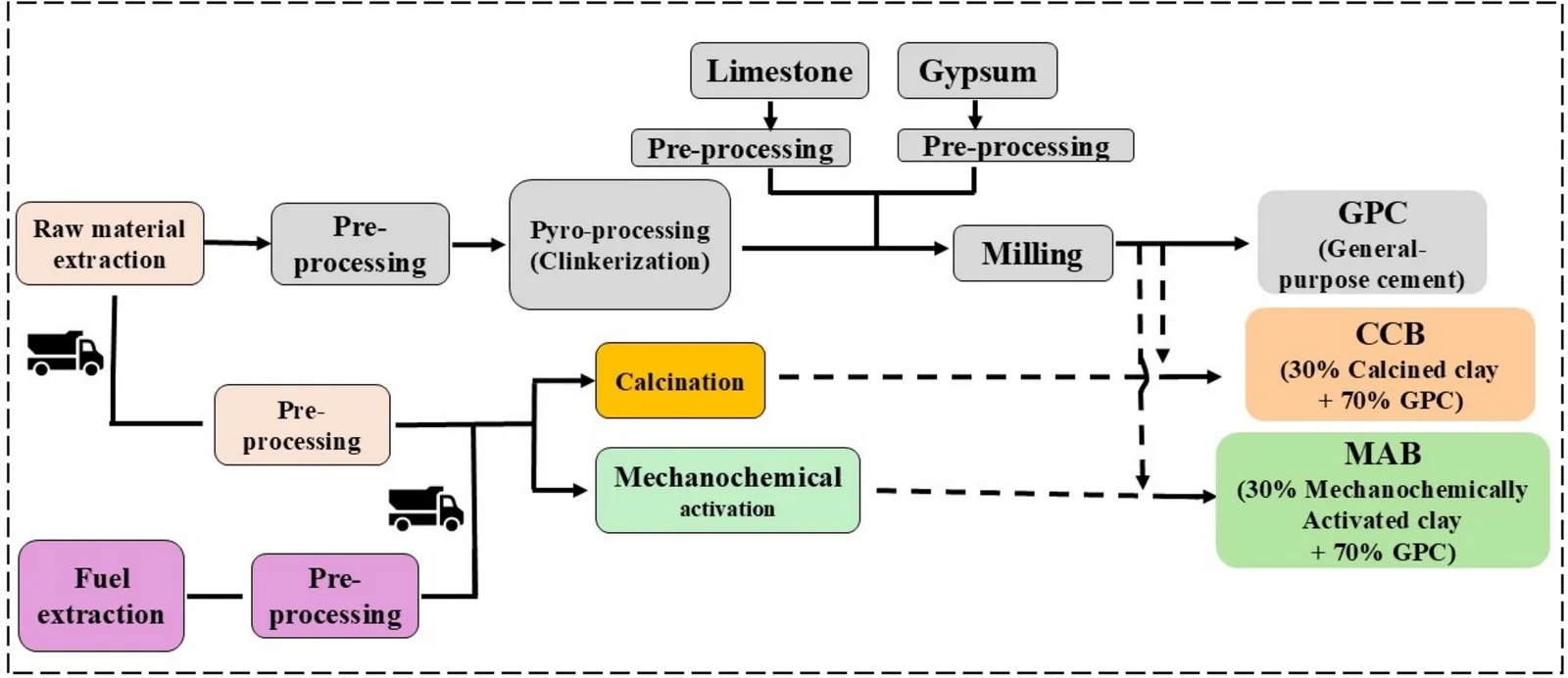
Boundaries of the LCA study (GPC, general-purpose cement; CCB, calcined clay-incorporated binders; MAB, mechanochemically activated clay-incorporated binders)

The LCA quantified and compared the environmental impact of incorporating calcined and mechanochemically activated clays as SCMs under Australian conditions. LCA was applied in a cradle-to-gate system boundary, from raw material extraction to binder production, where the binders are ready to be distributed from the cement plant in bulk form (Fig. 1). This includes all the emissions related to raw material extraction, transportation, pre-processing, activation, production of cement, production of binder from both clay and cement, and all the energy production-related emissions. The functional unit was defined as the environmental impact per 1 MPa of 28-day compressive strength, calculated using 1 kg of binder as the common reference mass. The same binder mass was used for all mixtures, but the 1 kg basis was adopted only to provide a consistent material comparison. The environmental impact calculated for 1 kg of each binder was divided by its measured mean 28-day compressive strength to obtain the impact per MPa. This enables an equivalent comparison between binders with different strengths.

#### Life cycle inventory

Life cycle impact calculations were performed using SimaPro v10.2.0.1. Primary data used in the analysis include compressive strength values measured from laboratory tests, directly obtained for the binder materials used in this study. This study utilized all the other secondary data from Ecoinvent (V 3.11) and AusLCI (V 1.45) (ALCAS 2020) databases (Table 4), as well as previous literature. AusLCI provides the region-specific data in this context.

**Table 4:** Inventory for the LCA study

Inventory list	Source(s)
General-purpose cement, Australian average/AU U	AusLCI
Cement plant/CH/I U/AusSD U	AusLCI, Ecoinvent
Industrial machine, heavy, unspecified, at plant/RER/I U/AusSD U	AusLCI, Ecoinvent
Lubricating oil, at plant/RER U/AusSD U	AusLCI
Electricity mix, Australia/AU U	AusLCI
Electricity, medium voltage, at grid/AT U/AusSD U	AusLCI
Electricity mix, 2050, Australia/AU U	AusLCI
Electricity, hydropower, at reservoir power plant, non alpine regions/RER U/AusSD U	AusLCI, Ecoinvent
Electricity, at wind power plant 2 MW, offshore/OCE U/AusSD U	AusLCI, Ecoinvent
Black coal, NSW, at mine {AU}| | AusLCI, U & Black coal, WA, at mine {AU}| | AusLCI, U	AusLCI
Diesel, at regional storage/RER U/AusSD U	AusLCI
Transport, truck, 40 t load {AU}| | AusLCI, U	AusLCI
Transport, lorry 20-28t, fleet average/CH U/AusSD/Link U	AusLCI
Transport, freight, rail, diesel/US U/AusSD U	AusLCI
Transport, transoceanic freight ship/OCE U/AusSD U	AusLCI
Heat, light fuel oil, at industrial furnace 1 MW/CH U/AusSD U	AusLCI, Ecoinvent
Light fuel oil, burned in boiler 100 kW, non-modulating/CH U/AusSD U	AusLCI, Ecoinvent
Water, unspecified natural origin, AU	AusLCI
Excavation, skid-steer loader/RER U/AusSD U	AusLCI
Excavation, hydraulic digger/RER U/AusSD U	AusLCI
Heat, district or industrial, other than natural gas {RoW}| heat production, straw, at furnace 300 kW | Cut-off, U	Ecoinvent
Natural gas, high pressure, Australia/AU U	AusLCI, Ecoinvent

Heat energy requirement for calcination was sourced from Ecoinvent for processes at 600 °C. A study by Tomatis et al. (2020) provides the values for 900 °C processing. All energy inputs were based on industrial-scale data and converted to align with Australian conditions. Specifically, the thermal energy for calcination was expressed as black coal consumption, using a conversion factor of 23.1 MJ/kg based on AusLCI data. Emissions to the air and the release of trace elements from coal combustion were calculated according to the Australian National Greenhouse Accounts Factors (Department of Climate Change 2023) and the study by Sloss (1995). The energy needed to activate the clay mechanochemically using a planetary ball mill was calculated using the equation (Eq. 1) proposed by Burgio et al. (1991) and later adopted by Ghayour et al. (2016). The parameters and their respective values employed in the calculations are presented in Table 5. The constant K depends on the ball geometry and was set to 1.5 in the original study.

**Table 5:** List of parameters and their values used in the calculation

Parameter	Value
Constant (*K*)	1.50E+00
Elasticity of collisions (*K*ₐ)	1.00E+00
Number of balls (*n*)	2.00E+02
Mass of each ball (*m*_b_) (kg)	4.00E-03
Plate spinning rate (*w*_p_) (rad s^−1^)	5.24E+01
Vial spinning rate (*w*_v_)	1.05E+02
Radius of the vial (*r*_v_) (m)	3.75E-02
Diameter of each ball (*d*_b_) (m)	1.00E-02
Distance from center of plate to vial center (*r*_p_) (m)	1.00E-01
Mass of powders used (*m*_p_) (kg)	8.00E-02
Volume of the vial (*V*_t_) (m^3^)	2.87E-04
Height of the vial (*H*_v_) (m)	6.50E-02
Volume of powders (*V*_p_) (m^3^)	8.89E-05

1 $$P =-\frac{1}{2\pi }\left(1-\varphi \right)K{K}_{a}n{m}_{b}\left({w}_{p}-{w}_{v}\right)\left(\frac{{w}_{v}^{3}\left({r}_{v}-\frac{{d}_{b}}{2}\right)}{{w}_{p}}+{w}_{v}{w}_{p}{r}_{p}\right)\left({r}_{v}-\frac{{d}_{b}}{2}\right)$$

The degree of filling of the vial (*φ*) was calculated using Eq. 2. It was assumed that this energy is the same for a large-scale ball mill.

2 $$\varphi =\frac{n{V}_{b}+{V}_{p}}{{V}_{t}}$$

Electricity inputs were modeled using the AusLCI database, which refers to the Australian Energy Update 2024 (Australian Government Department of Climate Change 2024) (Table 6).

**Table 6:** Electricity grid mix percentage

Fossil fuels				Renewables			
Black coal	Brown coal	Gas	Oil	Hydro	Wind	Bioenergy	Solar
35.0%	11.5%	17.8%	1.80%	6.1%	11.4%	1.1%	15.3%

Transportation of clay and coal was assumed to occur by road using diesel-powered trucks with a payload capacity of 40 tons, and operations inside the quarry were assumed to use a lorry with a 20–28 t capacity, with a fleet-average value. The calcination facility and the cement plant were located at New Berrima, NSW, Australia, for RSC, BS, CB, and HW. The cement plant in Kwinana was considered for BN clay. Google Maps was used to estimate all transport distances from this central location (Table 7). Limestone, used exclusively in the GPC mix, was sourced from local quarries and incorporated into the cement clinker during production. This includes the emissions from limestone extraction, transportation, and processing, as well as the CO₂ emissions associated with its use in clinker production. Limestone and sand were transported by rail and road using diesel-powered trucks (40 t), and part of the clinker was shipped from Japan and China. Clinker production was modeled using secondary inventory data from the selected AusLCI database, selected to represent Australian clinker production conditions. In addition to fuel combustion emissions, CO₂ emissions from the dehydroxylation of carbonate-containing clays were calculated separately and included in the overall life cycle analysis.

**Table 7:** Transport distances by mode for raw materials supplied to the cement plant

Description	Distance (km)		
	Road	Rail	Ship
Clays (RSC, BS, CB) to the cement plant	823		
HW clay to the cement plant	696		
BN clay to the cement plant	244		
Sand to the plant	55	63	
Limestone to the plant	55	63	
Imported clinker to the plant	75.4		8200

#### Life cycle impact assessment

This study utilized both the Australian Life Cycle Assessment Society (ALCAS) Best Practice LCIA Recommendations, Version 2.05, and ReCiPe 2016 Endpoint (H) (v.1.06). The method specified by ALCAS outlines a standardized approach for evaluating key environmental midpoint indicators specific to Australian conditions (Renouf et al. 2015). This framework enables converting inventory data into quantifiable environmental impacts across multiple categories. The indicators considered are GWP, ADM, ADF, ODP, POP, AP, EP, PMF, HTC, HTNC, FWT, HH, and WS. As this framework does not provide endpoint damage indicators, ReCiPe endpoint indicators were additionally used to support a broader interpretation of potential damage to human health, ecosystem quality, and resource scarcity. Therefore, the midpoint results provide the primary basis for detailed interpretation, while the endpoint results serve as supplementary indicators to understand broader environmental trends.

Global warming is quantified using the IPCC’s 100-year GWP model (Change 2007) expressed in kg CO₂ equivalents, in line with Australia’s national greenhouse gas inventory protocols. Guinée (2002) calculated abiotic depletion of elements using the CML-IA V4.8 (Aug 2016) method. Results are presented in kg Sb-eq, reflecting the resource scarcity based on known reserves and depletion rates. Fossil fuel depletion is similarly measured based on energy content (MJ), according to Guinée (2002) and CML-IA V4.8.

Water scarcity is assessed via the method proposed by Ridoutt and Pfister (2010), using regionalized water stress indices from Pfister et al. (2009), reported in m^3^ H₂O-eq. Eutrophication potential is based on the combined effects of nitrogen and phosphorus emissions, using CML-IA (Guinée 2002) adapted from Heijungs et al. (1992), with results in kg PO₄^3^⁻-eq. Acidification follows the characterization for critical load exceedance by Huijbregts et al (2000) and is also part of CML-IA V4.8, expressed in kg SO₂-eq.

Particulate matter formation is modeled through atmospheric dispersion and exposure using the CALPUFF model, following the study by Wolff (2000) under the TRACI v2.1 framework, in kg PM₂.₅-eq. Toxicity (human and freshwater) is estimated via the USEtox model, incorporating Australian regionalized characterization factors per Kounina et al. (2014), in Comparative Toxic Units (CTUh and CTUe). Photochemical ozone formation is measured using the Photochemical Ozone Creation Potential (POCP) method under CML-IA, in kg C₂H₄-eq.

For ozone depletion, values are based on the World Meteorological Organization (Douglass et al. 2011) using kg CFC-11-eq, compatible with the CML method. Ionizing radiation is assessed using the ILCD v1.0.9 (May 2016) framework, drawing from Frischknecht et al. (2000), in kBq U-235 eq. Each method is selected to ensure scientific robustness, international compatibility, and regional relevance for Australian LCAs.

Endpoint assessment was carried out using the ReCiPe 2016 endpoint (H). This evaluates environmental impacts across three areas: human health, ecosystem quality, and resource availability. Human health impacts are expressed in disability-adjusted life years (DALYs), representing the years of healthy life lost due to diseases, accidents, or other environmental burdens. Ecosystem damage is measured in species-years, indicating the potential loss of species over time due to environmental degradation. Resource scarcity is quantified in monetary terms (USD), reflecting the additional future costs required to extract minerals and fossil fuels as scarcity increases.

#### Sensitivity analysis

A sensitivity analysis was conducted to determine how parameter variation within the defined methodological framework affected the overall results of all endpoint indicators outlined in ReCiPe 2016. These endpoint results were used to compare broader environmental trends and should be interpreted as supplementary to the regionalized midpoint results. Specifically, this analysis includes transportation modes and changes in distance, heat production scenarios, and the electricity source. In the sensitivity analysis of transport modes, the transport distances of the clays were varied from their origin to 100 km, 250 km, and 500 km to assess the impact of varying distances.

Furthermore, various fuel sources were considered as potential thermal energy providers during calcination. The quantities needed to provide the same energy and resulting emissions were different. The fuel sources used are natural gas and heat generated through biomass burning. The electricity used for the thermal activation was compared with the proposed grid in 2050 and a decarbonized grid scenario using only hydropower and wind power.

## Results

The compressive strengths of the assessed binders (Table 3) range from 36.1 to 58.6 MPa. CC_RSC has the highest strength (58.6 MPa), the only mix to exceed the GPC strength. MA_BN has the lowest strength (36.1 MPa), which is 63% of the strength of GPC (57.5 MPa). All the calcined clay incorporated mixes exhibit higher strength than their respective mechanochemically activated clay incorporated mixes. Among the mechanochemically activated clay-incorporated binders, MA_RSC showed the highest strength of 53.8 MPa, which is 94% of the GPC strength. These results indicate clear differences in performance across the different binder systems, leading to varying environmental impacts per unit of performance.

Table 8 presents the environmental performance of all binder systems across the 13 impact categories. The strength results normalize all midpoint impact categories to impact per 1 MPa. Of the 13 midpoint categories, GWP and abiotic depletion are highlighted as the most critical impacts, showing the greatest differences between activation methods and carrying the greatest relevance for sustainability decisions.

**Table 8:** Midpoint life cycle impacts assessment

	Global warming (kg CO_2_ eq/MPa)	Abiotic depletion of elements (kg SB eq/MPa)	Abiotic depletion of fossil fuels (MJ NCV/MPa)	Ozone layer depletion (kg CFC-11 eq/MPa)	Photochemical oxidation (kg C2H4 eq/MPa)	Acidification (kg SO2 eq/MPa)	Eutrophication (kg PO4–- eq/MPa)	Particulate matter (kg PM2.5/MPa)	Human toxicity, cancer (CTUh/MPa)	Human toxicity, non-cancer (CTUh/MPa)	Freshwater ecotoxicity (CTUe/MPa)	Ionizing radiation (kBq U235 eq/MPa)	Water scarcity (m3 eq/MPa)
GPC	1.59E-02	2.38E-07	8.71E-02	8.65E-11	1.02E-06	3.99E-05	7.47E-06	2.10E-06	1.29E-11	3.22E-10	5.27E + 01	4.52E-05	1.36E-05
CC_RSC	1.29E-02	2.85E-07	8.50E-02	1.45E-10	9.27E-07	3.10E-05	6.03E-06	1.85E-06	1.68E-11	3.91E-10	5.32E + 01	3.15E-05	1.23E-05
CC_BS	1.38E-02	3.06E-07	9.11E-02	1.55E-10	9.93E-07	3.32E-05	6.46E-06	1.98E-06	1.80E-11	4.19E-10	5.70E + 01	3.37E-05	1.31E-05
CC_CB	1.46E-02	3.21E-07	9.58E-02	1.64E-10	1.04E-06	3.49E-05	6.79E-06	2.08E-06	1.90E-11	4.41E-10	6.00E + 01	3.55E-05	1.38E-05
CC_HW	1.48E-02	3.19E-07	9.68E-02	1.55E-10	1.15E-06	3.80E-05	6.91E-06	2.22E-06	1.85E-11	4.48E-10	6.10E + 01	3.64E-05	1.38E-05
CC_BN	1.35E-02	2.53E-07	8.48E-02	9.70E-11	9.80E-07	3.38E-05	6.16E-06	1.88E-06	1.35E-11	3.91E-10	5.31E + 01	3.26E-05	1.12E-05
MA_RSC	1.46E-02	6.10E-07	9.93E-02	1.54E-10	9.61E-07	3.49E-05	6.92E-06	2.08E-06	1.85E-11	3.63E-10	1.33E + 02	3.47E-05	1.54E-05
MA_BS	1.50E-02	6.26E-07	1.02E-01	1.58E-10	9.86E-07	3.58E-05	7.11E-06	2.14E-06	1.90E-11	3.72E-10	1.37E + 02	3.56E-05	1.58E-05
MA_CB	1.88E-02	7.83E-07	1.28E-01	1.97E-10	1.23E-06	4.48E-05	8.89E-06	2.67E-06	2.37E-11	4.66E-10	1.71E + 02	4.46E-05	1.97E-05
MA_HW	1.96E-02	8.08E-07	1.32E-01	1.92E-10	1.27E-06	4.65E-05	9.24E-06	2.76E-06	2.37E-11	4.83E-10	1.79E + 02	4.66E-05	2.02E-05
MA_BN	2.12E-02	8.47E-07	1.39E-01	1.53E-10	1.31E-06	5.02E-05	9.94E-06	2.88E-06	2.19E-11	5.17E-10	1.95E + 02	5.15E-05	2.07E-05

### Global warming potential

GWP measures the climate impact by converting all greenhouse-gas emissions into an equivalent amount of CO₂ (kg CO₂ eq) over 100 years. Almost all the calcined mixes show a marked reduction in GWP compared to GPC, indicating an environmental benefit from partial cement substitution. The magnitude of the reduction varies with the type of clay and the activation process. The GWP of mechanochemically activated clays is consistently higher than that of their calcined counterparts.

**Fig. 2:**
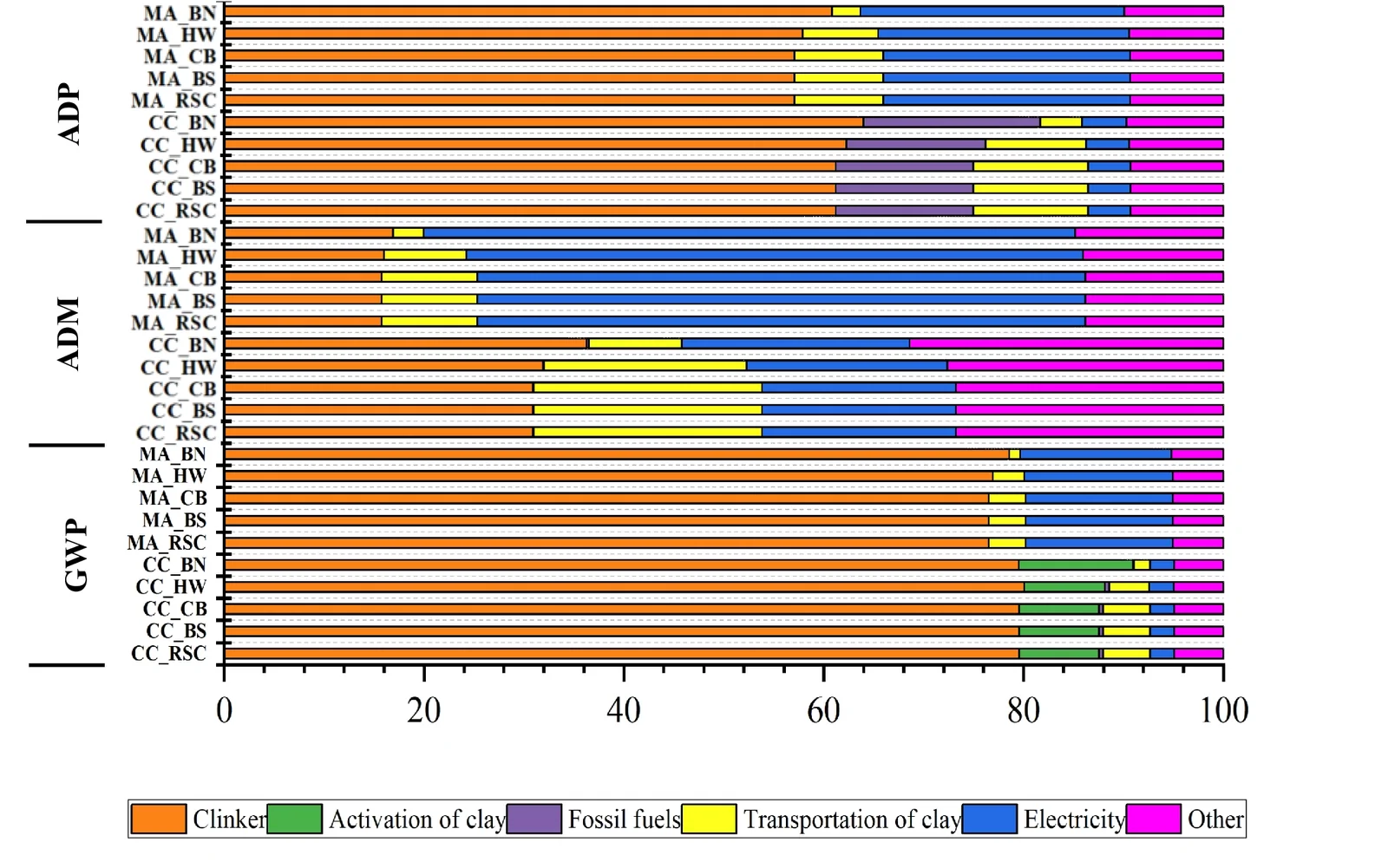
Contribution of materials and processes to GWP, ADM, and ADF of binders

RSC emerges as the most efficient alternative in both activation scenarios. The CC_RSC blend yields the lowest GWP (0.0129 kg CO₂ eq), representing an 18.6% reduction compared to GPC and a 10.8% reduction compared to MA_RSC. The 18.6% reduction in GWP of CC_RSC compared to GPC closely aligns with findings from previous studies on various types of calcined clays. For example, Dumani and Mapiravana (2024) reported a 17.3% reduction in carbon dioxide emissions when 30% of ordinary Portland cement was replaced with calcined clay. A study by Miller and Myers (2019) provides justification for this, finding a 17% reduction in GWP when 30% of cement is replaced with calcined clay. Bumanis et al. (2022) reported a 15% reduction in global warming potential when 25% waste kaolin was used as a cement replacement. Similarly, Sadok et al. (2022) found that a binder containing 25% calcined sediment and 75% cement reduced carbon dioxide production by 17.8%. The reduction observed in the present study falls within the range reported for calcined clay, waste kaolin, and calcined sediment systems, demonstrating that Australian natural mixed clays can achieve comparable environmental benefits despite their more variable mineralogical composition. Similarly, all the other calcined binders, along with MA_RSC and MA_BS, show substantial environmental benefits in terms of GWP. Figure 2 shows the contributions of different materials and activities to the GWP of 1 kg of activated clay, normalized to 1 MPa of binder strength.

Clinker production accounts for more than 75% of GWP across all binders. In CC_BN, the emission during the activation process is the major contributor to the GWP. This relates to the higher calcination temperature required for BN. The primary source of energy for the mechanochemical activation and the main contributor to the GWP is electricity. The contribution from electricity is higher than that in calcination, but mechanochemical activation has no direct atmospheric emissions. These emissions corresponds to the upstream emission of the electricity mix in Australia. In both activation methods, upstream emissions from fossil fuels have a negligible effect on GWP. The data indicate that calcination offers a robust pathway for reducing GWP across diverse clay types, with RSC and BN exhibiting the most favorable environmental profiles.

### Abiotic depletion of elements

Abiotic depletion of elements measures the use of non-renewable mineral resources by converting extracted metals and minerals into a standard unit of antimony equivalents (kg Sb eq). For mineral resource depletion, GPC registered a value of 2.38 × 10^−7^ kg Sb eq, serving as the benchmark for conventional production. All the binders had higher ADM values than GPC. Among all alternatives, MA_BN achieved the highest ADM, reflecting a 255% increase over GPC. Figure 2 shows that electricity is responsible for a significant fraction of ADM. Even though the energy used was the same for all the clays, the lower strength resulted in a higher value in the MA_BN binder.

The ADM of mechanochemically activated mixes generally surpasses that of calcined mixes. This illustrates the greater involvement of metals and minerals in electricity generation than in fossil fuels. MA_HW (8.08 × 10^−7^ kg Sb eq) records the most pronounced value among the mechanochemically activated mixes, followed by MA_BN (8.47 × 10^−7^ kg Sb eq). CC_RSC remains the binder with the lowest ADM of 2.85 × 10^−7^ kg Sb eq, but is 19.7% higher than GPC. Moreover, except for BN, the contribution of clinker to the ADM in calcined binders is less than the total contribution from the transportation of clay and the electricity used. In mechanochemically activated mixes, electricity used for the clay activation alone surpasses the contribution of clinker, which is 70% of the binder, while clay is only 30%.

### Abiotic depletion of fossil fuels

Abiotic depletion of fossil fuels, measured in MJ net calorific value (NCV), captures the non-renewable energy demand associated with each binder system (Guinée 2002). The impact stems from upstream stages in the life cycles of the fossil fuels used in all activities, including transportation. Notably, most calcined binders exceed even the GPC baseline (8.71 × 10^−2^ MJ NCV) in fossil fuel use. CC_HW reports the highest ADF among calcined alternatives at 9.68 × 10^−2^ MJ NCV with an 11.1% increase, while CC_BN is 8.48 × 10^−2^ MJ NCV, the lowest for a calcined binder. In contrast, mechanochemically activated binders demonstrate markedly higher fossil fuel dependence, with MA_BN (1.39 × 10^−1^ MJ NCV) and MA_HW (1.32 × 10^−1^ MJ NCV) showing increases of approximately 59.4% and 51.3%, respectively. More than 80% of the electricity grid is powered by electricity generated from fossil fuels (Table 6). This is why the values are higher, even without the direct use of fossil fuels in the activation process.

### Endpoint impact assessment using ReCiPe 2016

Table 9 illustrates the human health, ecosystem, and resource-related environmental impacts of all evaluated binder systems, normalized to 1 MPa.

**Table 9:** Endpoint life cycle impact assessment using ReCiPe 2016

	GPC	CC_RSC	CC_BS	CC_CB	CC_HW	CC_BN	MA_RSC	MA_BS	MA_CB	MA_HW	MA_BN
Human health	2.39E-08	1.94E-08	2.08E-08	2.19E-08	2.26E-08	2.02E-08	2.31E-08	2.37E-08	2.96E-08	3.08E-08	3.33E-08
Ecosystems	7.09E-11	5.67E-11	6.07E-11	6.39E-11	6.56E-11	5.89E-11	6.52E-11	6.69E-11	8.37E-11	8.72E-11	9.46E-11
Resources	2.96E-04	3.25E-04	3.48E-04	3.66E-04	3.61E-04	2.57E-04	3.58E-04	3.68E-04	4.60E-04	4.66E-04	4.52E-04

In the human health category, all calcined clay-based alternatives perform better than GPC, with an impact of 2.39 × 10^−8^ DALYs per functional unit. CC_RSC and CC_BN show the lowest impacts at 1.94 × 10^−8^ and 2.02 × 10^−8^ DALYs, respectively. Conversely, MA_BN shows the highest impact in this category at 3.33 × 10^−8^ DALYs, exceeding the GPC baseline by 39%.

Regarding ecosystem damage, a similar trend is observed. GPC cement exhibits an impact of 7.09 × 10^−11^ species-years, while all clay-based alternatives demonstrate lower values. The lowest ecosystem impact is associated with CC_RSC at 5.67 × 10^−11^, followed by CC_BN at 5.89 × 10^−11^, reinforcing the environmental advantage of using them. Binders such as MA_BN and MA_HW record values of 9.46 × 10^−11^ and 8.72 × 10^−11^ species-years, indicating a relatively higher ecological burden.

In the resource scarcity category, all the binders except CC_BN exceed the impact of GPC cement, which is 2.96 × 10^−4^ USD. The greatest impacts are observed for MA_HW (4.66 × 10^−4^) and MA_CB (4.60 × 10^−4^), resulting in 57.4% and 55.4% increases, respectively. This indicates a significantly higher future cost for extracting fossil and mineral resources associated with these compositions. In contrast, CC_BN shows a lower resource scarcity value of 2.57 × 10^−4^, a 13% reduction compared to the GPC baseline. These results highlight that selecting the raw material type, particularly recycled clays, is critical in reducing endpoint environmental impacts across all categories.

### Sensitivity analysis

#### Transportation distance

Local availability can reduce logistics burdens and environmental impact. Transportation involves different modes or combinations, comprising road, rail, and shipping. The endpoint impact assessment’s comparative results highlight the sensitivity to the transport distances. Table 10 illustrates the impact values for all the clays when the transport distance is fixed at 100 km, while keeping all the other distances involving the production of GPC the same.

**Table 10:** Endpoint life cycle impact assessment using ReCiPe 2016 for all the binders (transportation distance of clays is 100 km)

	CC_RSC	CC_BS	CC_CB	CC_HW	CC_BN	MA_RSC	MA_BS	MA_CB	MA_HW	MA_BN
Human health	1.85E-08	1.98E-08	2.08E-08	2.14E-08	2.01E-08	2.22E-08	2.28E-08	2.85E-08	2.98E-08	3.31E-08
Ecosystems	5.45E-11	5.84E-11	6.14E-11	6.31E-11	5.85E-11	6.31E-11	6.47E-11	8.10E-11	8.48E-11	9.40E-11
Resources	2.53E-04	2.71E-04	2.86E-04	2.93E-04	2.42E-04	2.90E-04	2.97E-04	3.72E-04	3.89E-04	4.31E-04

**Fig. 3:**
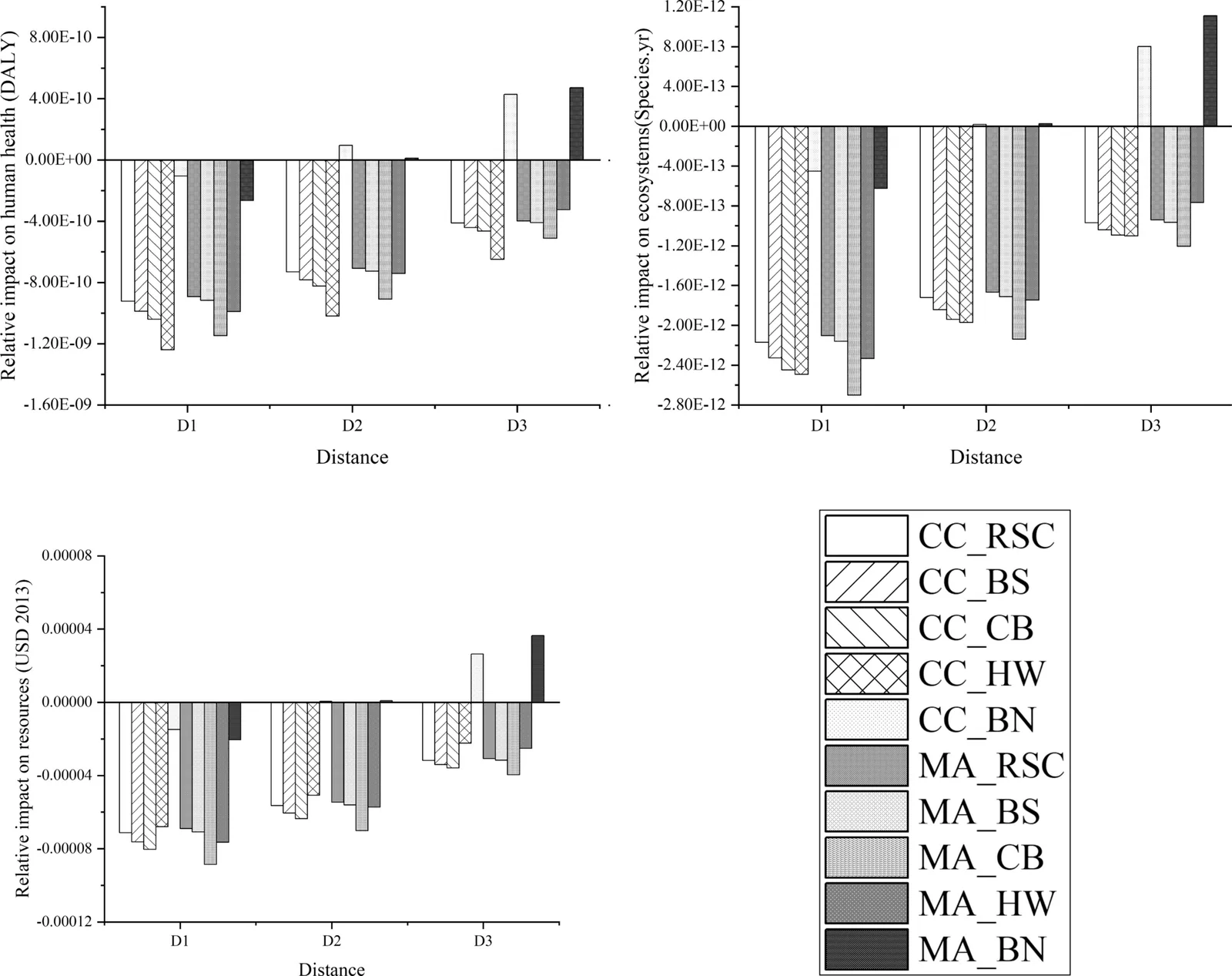
Comparison of the relative impact on endpoint impact categories at varying distances (D1 (100 km), D2 (250 km), D3 (500 km)), showing deviations from the original scenario

Figure 3 shows how changes in transportation distance affect the endpoint categories. The analysis indicates that transportation distance significantly affects all endpoint categories. Reducing the transportation distance for all clays to 100 km resulted in a significant decrease in impact values. CC_HW and MA_CB show the greatest decrease due to transportation-related variations in terms of their impact on human health and the ecosystem. CC_HW demonstrates a 5.5% reduction in the DALY value at D1, while MA_CB shows a 3.87% reduction.

Moreover, the impact on resources increased by 10.2% due to the increase in transportation of CC_BN to 500 km. In contrast, all the other clays experienced a reduction in distance, hence a reduction in endpoint categories. Restricting the transportation distance to 500 km decreased all categories, and further improvement was observed when it was reduced to 250 km. At 100 km, CC_RSC has the lowest values, reducing the total impact on human health from transportation from 12.6% to 7.8%. The 9.4% contribution to ecosystem-related impacts was reduced to 5.6%, and the 40.1% contribution to resource-related impacts was reduced to 18.2%. Among mechanochemically activated binders, MA_RSC shows the least impact when the transportation distance is restricted to 100 km. In the original scenario, transportation accounts for 10.6% of total effects on human health, 8.1% on the ecosystem, and 34.9% on resource-related categories. These values are reduced by 3.9%, 3.2%, and 19.2%, respectively, resulting in a reduction of 723 km in the transportation distance of clay.

#### Source of energy

**Fig. 4:**
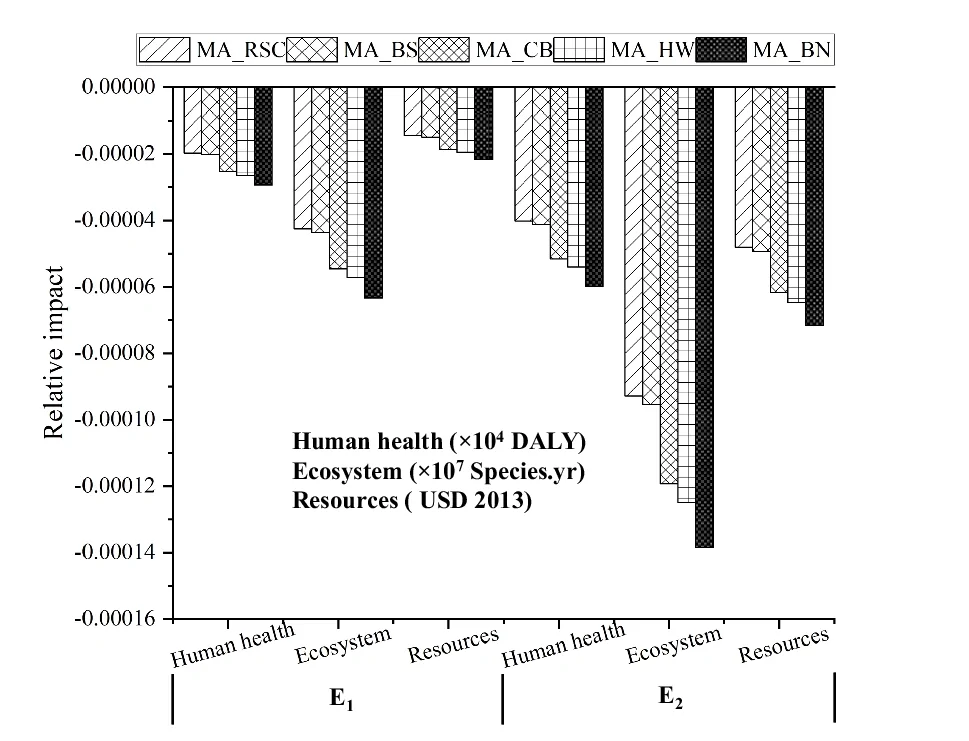
Comparison of the relative impact on endpoint impact categories with the use of various electricity sources for mechanochemical (E_1_ (proposed Australian electricity grid mix by 2050), E_2_ (decarbonized grid scenario)), showing deviations from the usual scenario

Another critical parameter is the energy source used in the activation process. This affects emissions during the activation process and all the associated upstream emissions. Figure 4 illustrates that the reductions in all categories align with the strength results. MA_RSC, having the highest strength, achieved the lowest impact values in all categories in the original scenario. The energy sources used during the process contribute 25.6% to human health, 21.9% to the ecosystem, and 55.2% to resource-related impacts. The transition to the projected 2050 electricity mix results in an 8.5% reduction in human health, a 6.5% reduction in ecosystem, and a 4.1% reduction in resource-related impacts for MA_RSC. All the mixes show similar reductions across all endpoint categories. The most significant improvements in MA_RSC include a 17.4% reduction in human health, a 14.3% reduction in the ecosystem, and a 13.4% reduction in resource-related impacts with decarbonized electricity.

**Fig. 5:**
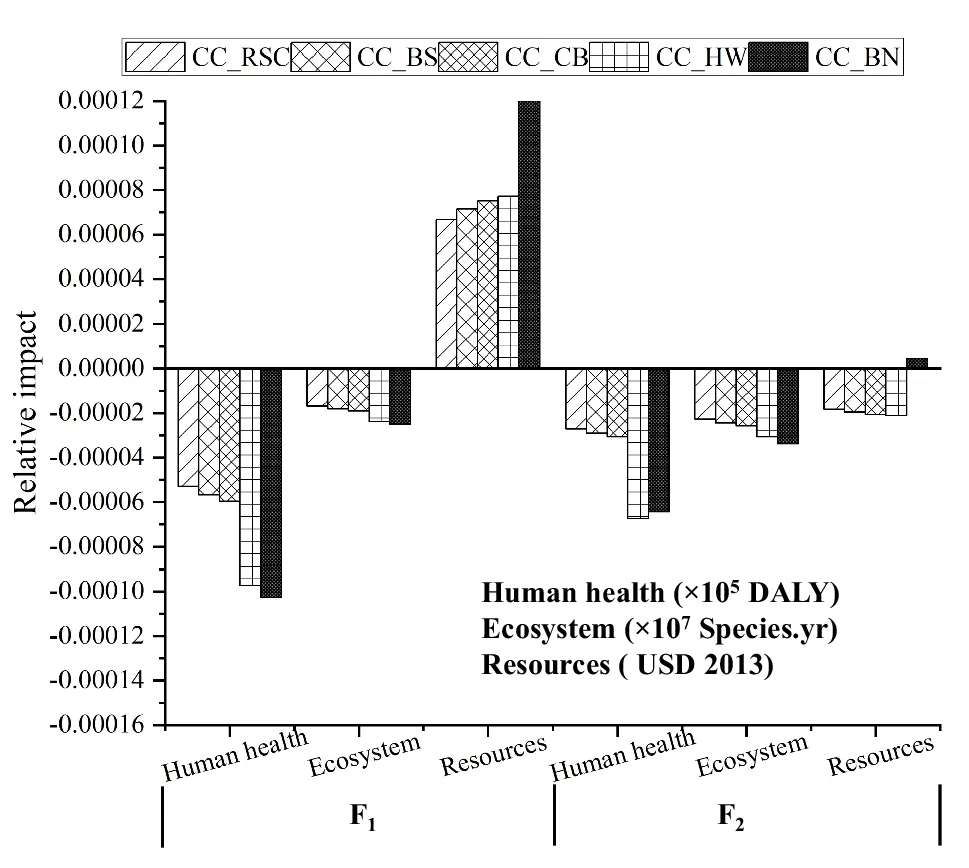
Comparison of the relative impact on endpoint impact categories with the use of various heat sources for calcination (F_1_ (natural gas), F_2_ (biomass)), showing deviations from the original scenario

Figure 5 illustrates the influence of fuel choice on the three endpoint impact categories for each clay type. Similarly, the fuel type used for heat production accounts for a considerable share of the binder's impacts. It accounts for 11.7% of the human health impact, 10.8% of the ecosystem impact, and 49.7% of the resource-related impact categories. In almost all scenarios, CC_BN achieves the highest reductions, but CC_RSC has the lowest impact in every category. The use of natural gas results in the greatest reduction in human health impacts, with CC_BN achieving a 5.1% decrease. The impact on the ecosystem is lowest when biomass is used, with a 5.7% decline observed in CC_BN. However, using natural gas leads to a substantial increase in resource impacts. CC_BN reports a 50.9% increase when natural gas is used. Biomass reduces the impact on resources for all other clays, except BN, which shows an increase of 1.7%.

## Discussion

### Change of impact with activation method

B_RSC emerges as the binder with the least environmental impact in both activation methods. However, a detailed examination of mechanochemical activation and calcination highlights critical environmental trade-offs. Calcination reduced GWP by up to 18.6% compared to GPC. This is consistent with previous studies (Bumanis et al. 2022; Dumani and Mapiravana 2024; Sadok et al. 2022). However, calcination increased abiotic depletion of fossil fuels by up to 11.1% compared to GPC. During calcination, fossil fuel combustion emits considerable direct CO₂ (Department of Climate Change 2023) and increases the ADF by consuming non-renewable fossil fuels. Combustion of coal releases trace elements (Furimsky 2000; Xu et al. 2004) into both air and soil, increasing toxicity, particulate matter, and ecosystem impacts (Hao et al. 2020; Odeh and Cockerill 2008; Tables 7 and 8). Using natural gas reduced the impact on human health and the ecosystem in CC_RSC by 2.7% and 3.0%, respectively, but increased resource burden by 20.6%. This increased the share of fuel type in the total impact on resources to 70.3%. However, the use of biomass balanced the reduction, reducing all three impacts by 1.4%, 4.0%, and 5.6% to 10.3%, 6.8%, and 44.1%, respectively.

On the other hand, the lower strength of the mechanochemically activated clay binders significantly increased their environmental impact per MPa. Mechanochemical activation relies heavily on electricity, thereby avoiding direct fossil-fuel combustion associated with calcination. Still, the reliance on fossil fuel-related power generation increased the ADF by up to 59.4%. Moreover, endpoint analysis using ReCiPe 2016 showed that mechanochemical activation increased human health burdens by up to 39% relative to GPC, while all calcined binders achieved lower health impacts than the baseline. Moreover, mechanochemically activated systems showed increased ADM, up to 255% higher than GPC in MA_BN, attributed primarily to electricity consumption and wear of the grinding media. Sensitivity analysis highlighted that the effectiveness of the process is inherently linked to the carbon intensity of the electricity grid used. Transitioning to a projected 2050 Australian grid reduced human health impact of MA_RSC by 8.5%, with a decarbonized grid offering up to 17.4% reduction across categories. This has changed the total share of energy sources to 8.2% for human health, 7.6% for the ecosystem, and 41.8% for resource-related impacts. This mirrors observations in the study by Sadok et al (2022), who reported that regional energy sourcing critically determines SCM sustainability.

Compared to calcination, mechanochemical activation of RSC and BS have the lowest impact across all three categories, when an alternative, greener energy source is available. This highlights that calcination offers greater strength and benefits primarily in terms of GWP. Mechanochemical activation amplifies the total impact unless paired with renewable energy sources. The results underscore the necessity for balanced optimization strategies to ensure sustainability without simply shifting environmental burdens.

### Impact of transportation

In all the binders, sea freight is the dominant mode, primarily due to the 23% imported clinker content in cement production, as specified in the AusLCI inventory. This aligns closely with the findings of Grant (2015) who reported a similar figure of 22.7%. However, for binders incorporating activated clays, the share of road-based transportation increases, reflecting the reliance on domestically sourced clays transported from regional deposits to production facilities.

BN, with the lowest proportion of road transportation, exhibits the second lowest environmental impact across nearly all categories in calcined binders (Table 8). Binders incorporating RSC, BS, CB, and HW clays exhibit similar transportation distances and lower activation energy in calcination. Except for CC_RSC, all other clays exhibit consistently higher environmental impacts across all midpoint indicators, which is notably linked to their similar, relatively high transport distances. CC_BN’s environmental advantages include higher strength and a shorter transportation distance. This is evident from Fig. 2, which highlights that transportation significantly contributes to ADF and ADM. Restricting transportation to the same distance for each clay type makes the picture clear. Both CC_BN and MA_BN have higher impacts than the original scenario with other binders.

CC_RSC and MA_RSC, with the lowest impact in both activation scenarios, exhibit 4.8% and 3.9% reductions in human health–related category, respectively, due to a 723 km reduction in transportation distance. These reduction values are 3.8% and 3.2% for ecosystem-related category and 21.9% and 19.2% for resource-related category. This implies that when using CC_RSC, the most effective binder, every increase of 100 km will result in an increase of 0.7% in human health, 0.5% in the ecosystem, and 3.0% in resource-related categories. For MA_RSC, these are 0.5%, 0.4%, and 2.7%.

Johansson et al (2024) found that road transportation can emit up to 32.5 times more CO₂ than rail and 2.24 times more than maritime shipping. These findings highlight the substantial emission burden associated with trucking, especially over long distances. In this context, rail transport is the most viable low-emission alternative, particularly for bulk construction materials. Therefore, while using locally sourced clays already offers clear environmental advantages, these benefits can be significantly amplified through integration with an efficient intermodal logistics network that leverages railways for long-haul movement and roads for local distribution.

### Key implications and trade-offs

These results illustrate the importance of adopting a multi-criteria perspective when evaluating binder sustainability. Although substituting 30% cement with activated clay consistently lowers the carbon footprint, it can also shift the burden to other impact categories. For example, most activated binders significantly reduced GWP relative to GPC. Yet, some mechanochemically activated binders’ net emissions are increased due to their lower strength. Likewise, while the mechanochemical activation route cuts GWP by using electricity, it leads to a higher relative ADM than the conventional mix. This underscores the risk of burden-shifting, where increased resource consumption offsets gains in climate or air quality metrics. Moreover, using natural gas to generate the heat needed for calcination instead of coal reduces the impact on human health and the ecosystem. However, the effect on resources increases by up to 50.9%.

These trade-offs highlight the need to optimize multiple aspects of the binder system simultaneously. This includes the raw material characteristics, activation method, energy source, and delivery logistics. These factors should be studied to maximize net environmental benefit in binder preparation. In practical terms, the most sustainable binder formulations should achieve a balanced reduction across key impacts, reducing impact on most mid-point categories. The findings suggest that partial cement replacement strategies should be tailored to regional conditions, based on the availability of reactive clays, renewable energy, and transportation infrastructure, to minimize potential trade-offs and fully capitalize on the environmental advantages of SCM use.

## Conclusion

This study conducted a performance-normalized life cycle assessment of five Australian clays activated by calcination and mechanochemical activation, replacing 30% of general-purpose cement. The environmental impacts of each system were evaluated across multiple categories using two methods (Australian Life Cycle Assessment Society (ALCAS) Best Practice LCIA Recommendations, Version 2.05, and ReCiPe 2016 Endpoint (H) (v.1.06)) to identify the most sustainable activation pathway under region-specific conditions. The key findings of the study are as follows.

- Calcined low-grade clays incorporated binders reduced global warming potential by 18.6% compared to general-purpose cement, outperforming mechanochemically activated clays incorporated binders by up to 64.4% under the assessed Australian energy mix.
- Mechanochemically activated clays increased abiotic depletion potential by 255% due to a substantial reliance on electricity and grinding media.
- Reducing transport distances by 100 km could decrease impacts on resources by 3%, human health by 0.7%, and ecosystems by 0.5%.
- Employing renewable energy sources for mechanochemical activation could decrease endpoint environmental impacts by up to 18%, and biomass as fuel for calcination resulted in up to 6% reduction.
- Calcination is the more environmentally favorable activation route for Australian natural mixed clays under the assessed conditions. However, mechanochemical activation has the potential to become more environmentally competitive with the use of low-carbon electricity and optimized milling energy demand.
- Burden shifting was observed, where improvements in climate-related impacts (e.g., up to 7.8% GWP reduction) were counterbalanced by increases in other categories (e.g., up to 156% rise in ADM).

## Data Availability

Data will be made available on reasonable request.

## Abbreviations

GPC: General-purpose cement
RSC: Red sealing clay
BS: Blended sealing clay
CB: Clay B
HW: Hanson white clay
BN: Bentonite clay
CC_(Clay name): Binder with 30% calcined clay + 70% cement
MA_(Clay name): Binder with 30% mechanochemically activated clay + 70% cement
B_(Clay name): Binder with 30% activated clay (any method) + 70% cement
CCB: Calcined clay incorporated binders
MAB: Mechanochemically activated clay-incorporated binders
GWP: Global warming potential
ADM: Abiotic depletion potential of elements
ADF: Abiotic depletion potential of fossil fuels
ODP: Ozone layer depletion potential
POP: Photochemical oxidation potential
AP: Acidification potential
EP: Eutrophication potential
PMF: Particulate matter formation
HTC: Human toxicity (cancer)
HTNC: Human toxicity (non-cancer)
FWT: Freshwater ecotoxicity
HH: Ionizing radiation
WS: Water scarcity
